# Efficacy of endotracheal intubation in helicopter cabin vs. ground: a systematic review and meta-analysis

**DOI:** 10.1186/s13049-024-01213-1

**Published:** 2024-05-10

**Authors:** Lydia Johnson Kolaparambil Varghese, Jan-Jakob Völlering, Edoardo De Robertis, Jochen Hinkelbein, Jan Schmitz, Tobias Warnecke

**Affiliations:** 1 University Department of Anaesthesiology, Intensive Care Medicine, and Emergency Medicine, Johannes Wesling Klinikum Minden, University Hospital Ruhr-University Bochum, Minden, Germany; 2 European Society of Aerospace Medicine (ESAM), Cologne, Germany; 3 Department of Mathematics and Informatics, University of Osnabrück, Osnabrück, Germany; 4 Division of Anaesthesia, Analgesia, and Intensive Care, Department of Medicine and Surgery, University of Perugia, Perugia, Italy; 5 Department of Anaesthesiology and Intensive Care Medicine, University Hospital of Cologne, Cologne, Germany; 6 Department of Sleep and Human Factors Research, German Aerospace Centre, Cologne, Germany; 7 Intensive Care, Emergency Medicine, and Pain Therapy, University Clinic of Anaesthesiology, Klinikum Oldenburg, Oldenburg, Germany

**Keywords:** Endotracheal intubation, Systematic review, Meta-analysis, Helicopter, Ground, First pass success rate, HEMS, Time of intubation

## Abstract

**Background:**

Pre-hospital endotracheal intubation (ETI) is a sophisticated procedure with a comparatively high failure rate. Especially, ETI in confined spaces may result in higher difficulty, longer times, and a higher failure rate. This study analyses if Helicopter Emergency Medical Services (HEMS) intubation (time-to) success are influenced by noise, light, and restricted space in comparison to ground intubation. Available literature reporting these parameters was very limited, thus the reported differences between ETI in helicopter vs. ground by confronting parameters such as time to secure airway, first pass success rate and Cormack-Lehane Score were analysed.

**Methods:**

A systematic review and meta-analysis were conducted using PUBMED, EMBASE, Cochrane Library, and Ovid on October 15th, 2022. The database search provided 2322 studies and 6 studies met inclusion and quality criteria. The research was registered with the International Prospective Register of Systematic Reviews (CRD42022361793).

**Results:**

A total of six studies were selected and analysed as part of the systematic review and meta-analysis. The first pass success rate of ETI was more likely to fail in the helicopter setting as compared to the ground (82,4% vs. 87,3%), but the final success rate was similar between the two settings (96,8% vs. 97,8%). The success rate of intubation in literature was reported higher in physician-staffed HEMS than in paramedic-staffed HEMS. The impact of aircraft type and location inside the vehicle on intubation success rates was inconclusive across studies. The meta-analysis revealed inconsistent results for the mean duration of intubation, with one study reporting shorter intubation times in helicopters (13,0s vs.15,5s), another reporting no significant differences (16,5s vs. 16,8s), and a third reporting longer intubation times in helicopters (16,1s vs. 15,0s).

**Conclusion:**

Further research is needed to assess the impact of environmental factors on the quality of ETI on HEMS. While the success rate of endotracheal intubation in helicopters vs. on the ground is not significantly different, the duration and time to secure the airway, and Cormack-Lehane Score may be influenced by environmental factors. However, the limited number of studies reporting on these factors highlights the need for further research in this area.

## Background

In the past five decades, helicopter emergency medical services (HEMS) have been playing a cardinal role in the pre-hospital critical emergency care setting [1]. As hypoxia can lead to rapid deterioration, professional and rapid response from HEMS with advanced skills of airway management is required. In this context, pre-hospital endotracheal intubation (ETI) is considered the gold standard in airway management [2].

The efficacy of HEMS in comparison to ground emergency medical services (GEMS) has been evaluated in several studies, as well as the grade of difficulty of pre-hospital intubation in comparison to in-hospital intubations [3]. The HEMS seems to be crucial in the transport of trauma patients in particular areas such as wilderness, mountain regions, or rural areas, resulting in reduced rescue time, widening the range of transport and improving the patient survival outcome [4]. Patients with traumatic brain injury rescued by HEMS, seem to be associated with better vital parameters such as oxygenation, ventilation, and blood pressure [5].

Endotracheal intubation (ETI) is a complex procedure that involves several steps, such as pre-oxygenation, endotracheal tube preparation, intravenous access establishment, and administration of induction medications [6]. Airway management inside the helicopter can often be more challenging than on the ground due to the different environmental factors that can affect the quality of the manoeuvre. These factors include noise and vibration of the helicopter, limited cabin space, and the more critical patients and complex medical conditions typically encountered in HEMS [2]. Other factors that could impact endotracheal intubation quality might include the intubator’s anthropometrics and the patient’s head reclination. Normally, in-flight intubation is avoided to reduce the impact of environmental factors, but pre-take-off intubation may affect the total prehospital time. This time delay can potentially lead to worsening outcome in some patients.

Despite the many studies examining endotracheal intubation in HEMS, there is still a significant research gap when it comes to understanding the impact of environmental factors and other variables on the quality and efficacy of this procedure. Although numerous case reports, surveys, and small observational studies have reported positive results, the overall success rate of pre-hospital intubations has been reported to range between 77 and 100% with a better outcome in physician HEMS ETI [7, 8]. To our knowledge, no systematic review has yet been conducted to evaluate the efficacy of ETI in HEMS.

The main parameters that were taken into consideration in this study are the duration of intubation, time to secure the airway, first-pass intubation success rate and Cormack-Lehane scores. First-pass intubation success is a critical component of effective prehospital airway management [9]. The success of the initial intubation attempt can significantly influence patient outcomes by minimizing the risk of complications such as hypoxia and aspiration and reducing the need for additional attempts, which may increase patient discomfort and the likelihood of adverse events [10]. Therefore, evaluating the efficacy of endotracheal intubation in both helicopter and ground transport settings is of paramount importance in improving prehospital airway management practices. We hypothesize that the above-mentioned parameters are prolonged and/or worse in helicopter cabin endotracheal intubation.

This study aims to address the gaps in the existing literature by comparing the first-pass success rates of endotracheal intubation in HEMS and ground stretcher settings. We conducted a systematic review and meta-analysis of current research to provide comprehensive insights into the efficacy of endotracheal intubation in these transport settings. By synthesizing the latest evidence, we aim to contribute to the advancement of prehospital airway management and ultimately improve patient outcomes.

Our aim is to provide insight to the undermentioned questions:

(i) How do environmental factors such as noise, vibration, and daylight influence the quality of ETI in HEMS? (ii) How do the intubator’s anthropometrics and patient head reclination angle affect the success of the procedure? (iii) What are the differences in ETI success rates between a helicopter and a ground stretcher?

## Methods

### Protocol and registration

PRISMA 2020 guidelines (Preferred Reporting Items for Systematic Reviews and Meta-Analyses) criteria were followed in the implementation of this research [11].

The search approach, study selection, bias evaluation, and data extraction and analysis methods were predetermined.

The study is registered with PROSPERO International Prospective Register of Systematic Reviews under the number CRD42022361793. The relative protocol can be accessed on the PROSPERO database.

### Eligibility criteria

This systematic review included comparative, retrospective, descriptive, and prospective studies that reported Endotracheal Tracheal Intubation (ETI) as the main intubation technique in Helicopter Emergency Medical Services (HEMS) for non-paediatric patients. For the Randomised Control Trials (RCTs) included in the meta-analysis, the comparability between the parameters on the helicopter and the ground was a fundamental inclusion criterion. Due to the infeasibility of recruiting patients in an emergency context RCT, the population evaluated in the meta-analysis was exclusively mannequins. Additionally, we only accepted studies with full text in English and did not restrict by the year of publication. As ETI is our preferred airway management technique, any studies reporting any technique other than ETI were excluded from the study, as reported in Box 1.

**BOX 1** Criteria for Study Selection: Inclusion and Exclusion Criteria for the study inclusion.

**Table:** 

Inclusion criteria
1) Adult patients
2) Endotracheal intubation as the technique of choice
3) English literature
4) Presence of a comparing group (Ground vs. HEMS)
*Exclusion criteria*
1) Paediatric patients
2) Other airway management techniques

### Search strategy

Cochrane Library, Ovid, EMBASE and PubMed were the sources of predilection and were last consulted in October 2022 without any restrictions. For PubMed, the search strategy was: “Airway Management AND Aircraft”; “Airway Management AND Helicopter”; “Intubation AND Aircraft”; “Helicopter AND Intubation”; “Ground intubation AND Helicopter intubation”. An identical search strategy was employed across all the other search engines. The approach was modified later as the aircraft cabin airway management results were not in line with our research project objective. Moreover, relevant publications’ bibliography was examined for additional screening of eligible articles.

### Selection process

Rayyan [12] was utilised in the process of study selection. JH, TW, JS and LJKV have screened the titles and abstract, double-blinded to avoid selection bias. The reviewers approached the screening process with a collaborative mindset, working together to ensure that each step was completed efficiently and accurately. Any disagreements that arose between the screeners were effectively resolved through the involvement of the third author, who served as a mediator. Despite the occasional disagreement, the eligibility criteria remained transparent and straightforward throughout the process. Additionally, to streamline the screening process, any duplicate entries were removed prior to the second screening stage. Overall, the reviewers maintained a rigorous and thorough approach to screening, resulting in high-quality and reliable data.

For entries in which relevance could not be determined based on the title and abstract alone, a second eligibility check was conducted by reviewing the full text. The citation manager used in all the steps of this study is Zotero® (George Mason University, Fairfax, United States of America).

### Data collection process and data items

Using Microsoft Excel® (Microsoft corp., Redmond, United States of America), each study, which passed the screening test, was tubulated to better envision the parameters and settings accordingly. The information included for each study were:

i) Study characteristics, population size, type of population (Mannequin vs. Human) and the intubation performer (ER physician vs. non-physician).
ii) Setting specifying the type of Helicopter and the status of the helicopter (en-route vs. static).
iii) parameters such as duration of intubation, time to secure airway, first pass success, and Cormack-Lehane score; iv) outcome of parameters compared and/or observed in the study.

For the meta-analysis, the parameter extrapolated from the RCTs were the median duration of intubation on the helicopter compared to the ground, the time to secure the airway on the helicopter vs. ground, the first-pass success rate in the helicopter vs. ground, and Cormack-Lehane in the helicopter vs. ground. In the settings, it specified whether it was a ground stretcher, or an emergency room employed as the control location.

### Assessing risk of bias and study quality

Cochrane Collaboration’s tool Rob2 was utilised for assessing the risk of bias in the included RCTs [13] to appraise the internal validity of the studies. Numerical scales were avoided as Cochrane reports that there is no basis for weighing different items with them.

Studies are allocated in “low,” “some concerns,” or “high” risk of bias through the usage of this tool. Most of the studies reported a risk of bias between “low” and “some concerns” in all five domains. The main reason to report some domains as “some concerns” is due to the impossibility of blinding the ETI performer and mannequins. Thus, the RCT analysed could be overall considered as low bias. All the RCTs with low bias were included in the meta-analysis.

The ROBINS-I was for the assessment of the study quality of the observational studies included in the study [14].

### Data synthesis and statistical analysis

Meta-analysis was performed on the data extracted from eligible studies using JASP (University of, Amsterdam, The Netherlands) [15]. Pooled effect sizes were reported as mean differences (MD), with corresponding 95% confidence intervals (CI). Heterogeneity was assessed using the I2 statistic. An I2 value of 0–50% was considered to represent homogeneity, 50–90% denotes substantial heterogeneity, and 90–100% high heterogeneity. A restricted-effects model was used to estimate pooled effect sizes for the meta-analysis to account for anticipated clinical diversity and methodological variability among studies.

Publication bias was evaluated by visually examining the funnel plot for asymmetry.

The quality of evidence for each outcome was assessed using the Grading of Recommendations, Assessment, Development, and Evaluations (GRADE) approach, and the results were summarized in a GRADE evidence profile. The GRADE approach evaluates the quality of evidence based on five factors: study design, risk of bias, inconsistency, indirectness, and imprecision. The quality of each study was categorized as high, moderate, low, or very low.

## Results

### Study selection

**Fig. 1:**
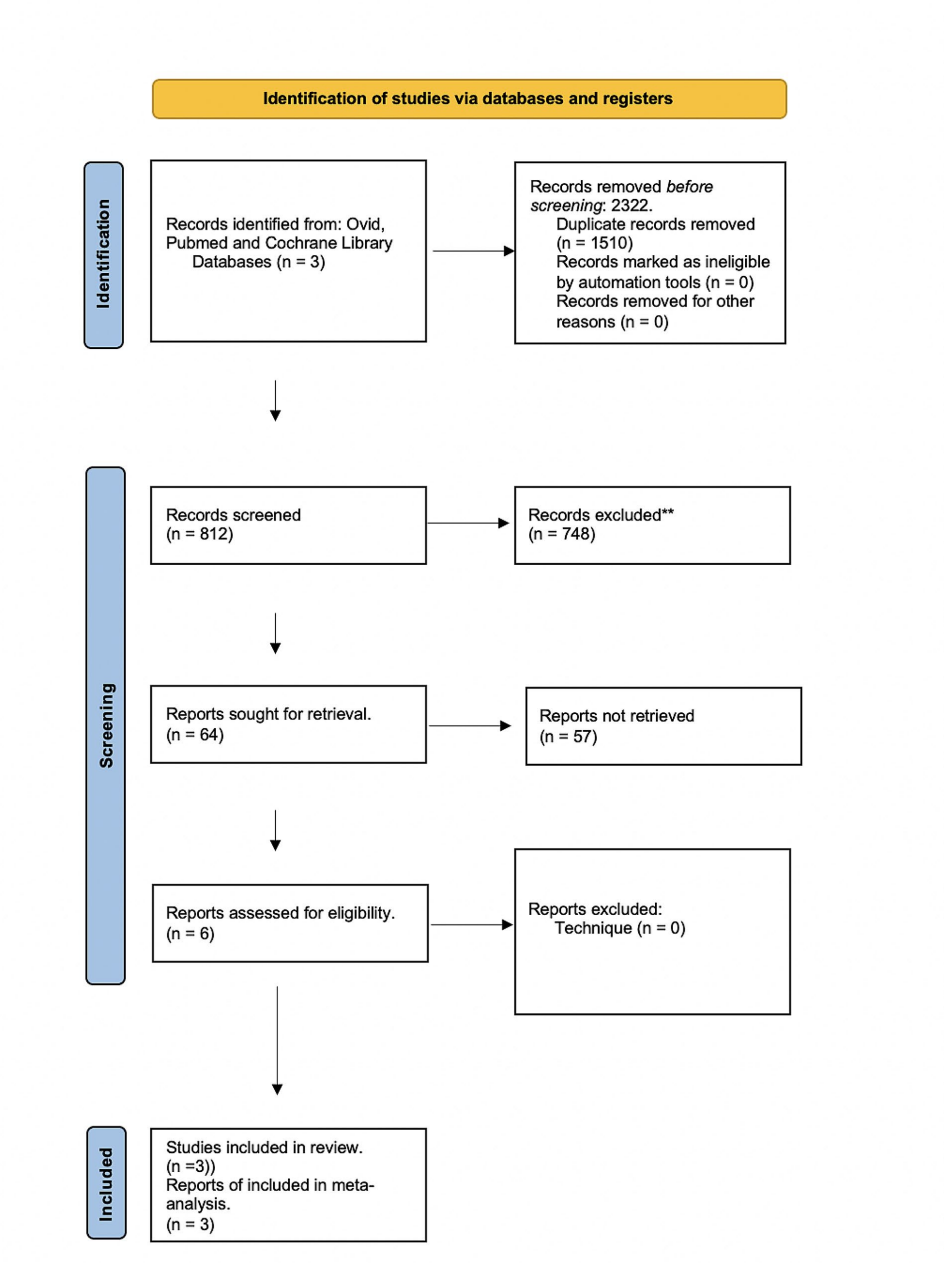
Prisma flowchart

We conducted a comprehensive search of Ovid, PubMed, EMBASE and Cochrane Library, which initially yielded 2322 potential studies. After removing duplicates, we screened 812 studies based on their titles and abstracts. Out of these, 748 studies were eliminated as they failed to meet the inclusion criteria. The remaining 64 studies underwent full-text screening, and 57 of them were excluded due to inadequate technique or lack of sufficient data. Ultimately, we included 3 observational studies in our systematic review and 3 randomized controlled trials (RCTs) in our meta-analysis, following the Preferred Reporting Items for Systematic Reviews and Meta-Analyses (PRISMA) guidelines (Fig. 1).

### Study characteristics

The systematic review and meta-analysis included 6 studies in total, consisting of 3 randomized controlled trials (RCTs) and 3 observational studies. The studies were published between 1992 and 2021 and were conducted in various countries across different continents. Sample sizes ranged from 43 to 376 patients, with a total of 107 patients included in the meta-analysis. Most of the studies were retrospective or comparative in design.

The three observational studies included in the systematic review were conducted to investigate the effectiveness of in-flight intubation in comparison to ground intubation for critically ill patients requiring airway management during aeromedical transport. The studies were conducted in different countries (The United States of America, the United Kingdom and Japan) and included mannequins. The study characteristics of the ones included in the systematic review are reported in Table 1.

**Table 1:** Characteristics of the relevant studies included in the systematic review

Author	Year	Study method	Helicopter	Study subject	Sample number	Number of Intubators	Setting
Stone et al.	1994	Comparative	BO-105 helicopter	Mannequin	30	N.A	In-flight
Harrison et al.	1997	Retrospective cohort studies	BK-117	Human	303	N.A	In-flight
Maeyama	2020	Retrospective cohort studies	EC 135	Human	376	N.A.	In-flight

The meta-analysis included three RCTs that were conducted to compare the first pass attempt, intubation time and difficulty of intubation outcomes of helicopter ETI versus ground ETI. The studies employed endotracheal intubation to measure the effectiveness of an in-flight helicopter intubation and ground intubation, which enabled us to combine the results in a meta-analysis. The meta-analysis allowed us to generate more precise estimates of the effectiveness of ETI, as well as to explore the sources of variability between the studies. The study characteristics of the RCTs included in the meta-analysis are reported in Table 2.

**Table 2:** Characteristics of the relevant studies included in the meta-analysis

Author	Year	Study Method	Helicopter	Study subject	Sample number	Number of Intubators	Setting
Gellerfors et al.	2015	RCT	Black Hawk UH60M	Mannequin	18	12	Stationary on ground
Kornhall et al.	2018	RCT	H145 HEMS	Mannequin	14	14	Stationary on ground
Lepa et al.	2021	RCT	EC135 Helicopter	Mannequin	75	15	Stationary on ground

### Quality Assessment

The quality of the studies included in this review was assessed using the Rob2 tool [13], which is a widely used tool for assessing the risk of bias in randomized controlled trials (RCTs) and non-randomized studies, developed by the Cochrane Collaboration. The assessment of the quality of each study was conducted by LKVJ and JJV, and any discrepancies were resolved through discussion and consensus of JH.

The Rob2 tool is used to assess the quality of research studies by analysing potential sources of bias such as randomization procedures, deviations from intended interventions, missing outcome data, measurement of outcomes, and selection of reported results. By evaluating these potential sources of bias, the Rob2 tool provides a comprehensive approach to assessing the quality of research studies and minimizing the risk of bias influencing study outcomes. Each study is assigned a rating of high, moderate, or low quality to the study.

The results of the quality assessment show that one of the studies was of high quality, two were of moderate quality, and none were of low quality.

Overall, the quality of the studies included in this review was moderate, indicating that the results should be interpreted with some caution.

### Study characteristics in the meta-analysis: a descriptive summary

The meta-analysis was conducted on three randomised control studies. The studies were performed in H145HEMS, EC135 Helicopter and Black Hawk UH60M ambulance helicopters. Only Gellerfors et al. [16] considered the night vision vs. daylight vision impact on the ETI success, but for meta-analysis purposes, it was not taken into consideration and will be reported in the discussion part. All three studies have shown a 100% total success rate.

Interestingly, these studies came to highly different conclusions regarding the mean time of endotracheal intubation between helicopters and ground settings. Kornhall et al. [17] reported that the ETI in the helicopter setting was found to be notably faster than on the ground (13,0s vs. 15.5 s), while Gellerfors et al. stated no noticeable difference in the intubation time between the two settings (16.5s vs. 16.8s). Lepa et al. [18] had a larger sample size of 75, and they reported the mean time of intubation in the helicopter to take approximately 7% longer than on the ground (Table 3).

Despite the contrasting results of the smaller sample-sized studies, the larger study provides more reliable and valid data due to its larger sample size. This study also had the highest quality score based on the Rob2 scale.

Overall, these results suggest that while the time taken for endotracheal intubation varies between helicopter and ground settings, there is no clear consensus in the literature. It is important to consider the quality of the studies and their sample sizes when interpreting the results, as a matter of fact, the total reported in the Table 1 is weighted with the sample size itself. It is important to also note that while these differences reached statistical significance, their clinical significance may be limited. For example, a mean difference of 3.5 s in intubation time, while statistically significant, may not have a meaningful impact on patient outcomes or other pragmatic considerations in clinical practice. These findings underscore the importance of considering both statistical significance and clinical relevance when interpreting study results.

**Table 3:** Study characteristics in the Meta-Analysis: Median and mean duration of intubation on ground vs. helicopter

	Median Duration of Intubation in Helicopter (s)	Median Duration of Intubation on Ground (s)	Mean Difference in Duration of Intubation (s)	Relative Difference in Intubation time in the helicopter vs. ground
Kornhall et al., 2018	13,0	15,5	-2,5	-16,1%
Lepa et al., 2021	16,1	15,0	1,0	7,2%
Gellerfors et al., 2015	16,5	16,8	-0,3	-1,7%
Total	16,3	15,9	0,4	2.7%

### Meta-analysis results

**Fig. 2:**
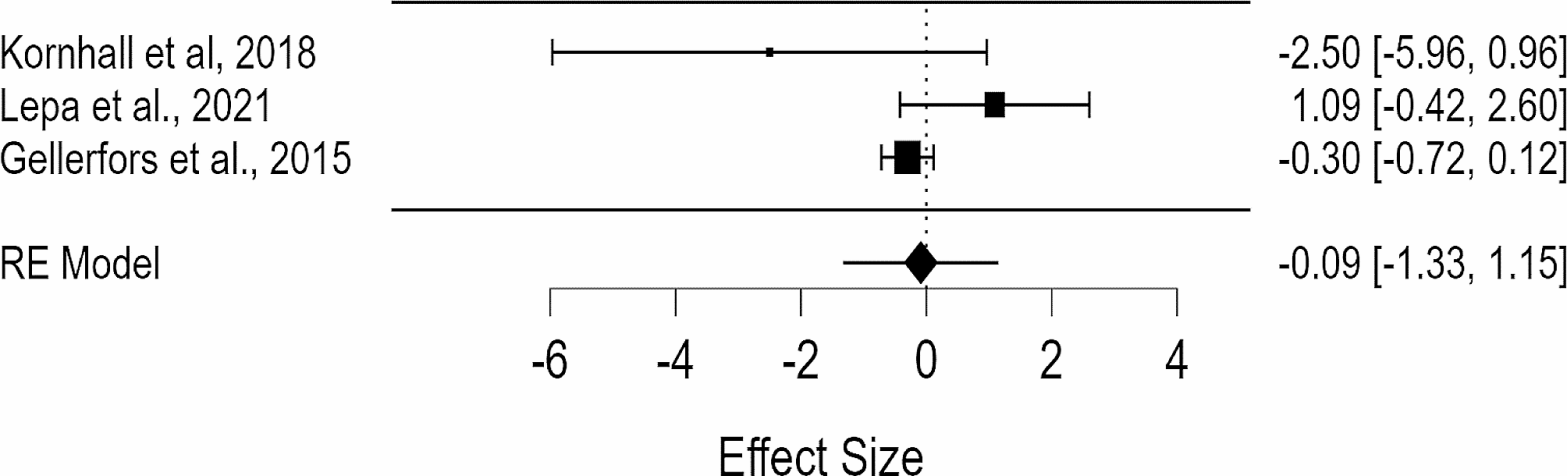
Forest plot

**Fig. 3:**
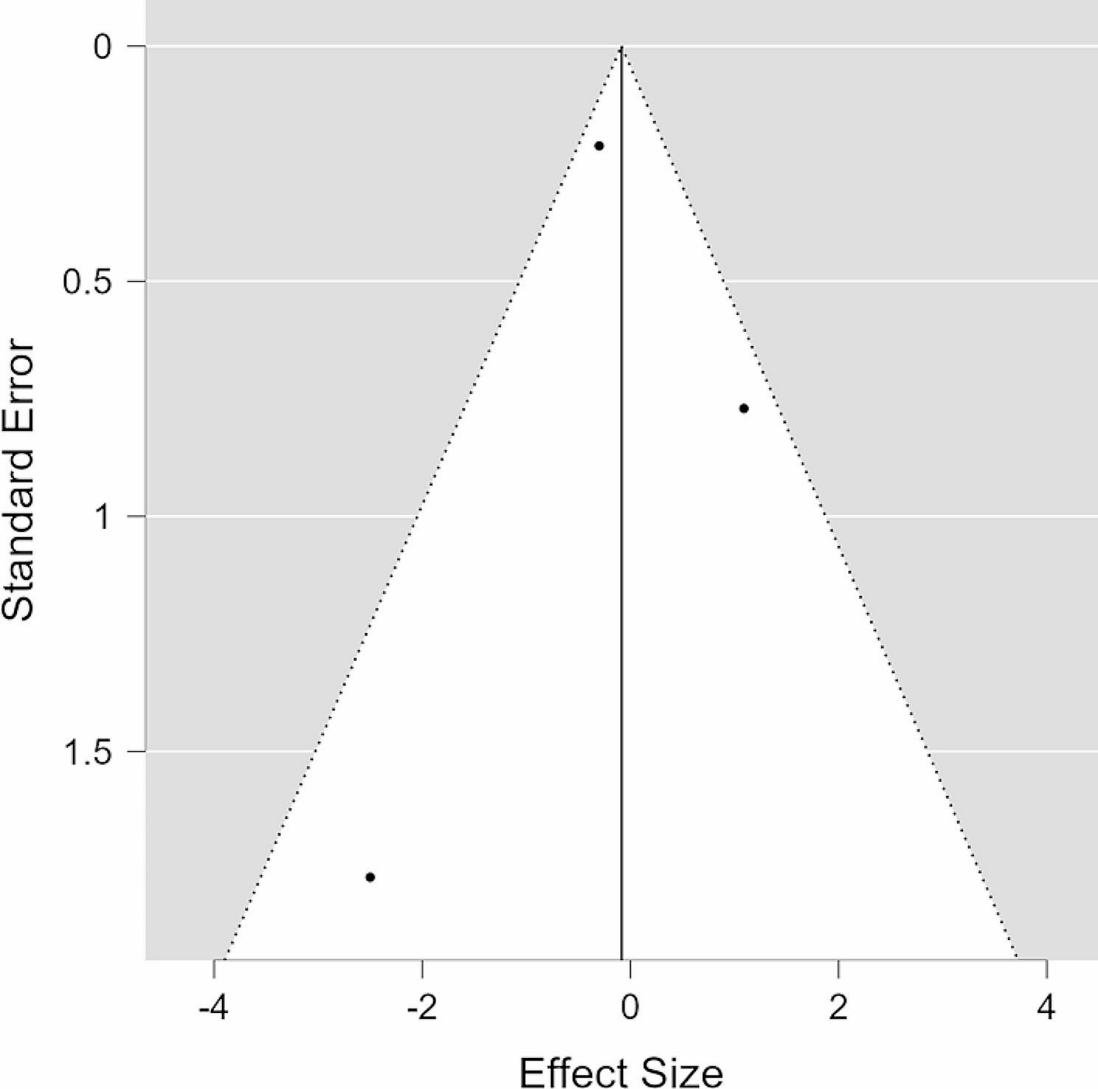
Funnel plot

Both the forest plot and funnel plot were used to graphically represent the “mean difference in duration of intubation (DOI)” in seconds to the standard error for each study, utilizing data points from the original Table 3. In the forest plot, the black dotted line represents the null line, and it is evident that Lepa et al. was the only study to the right of this line, indicating that intubation time was prolonged in the helicopter compared to the ground (Fig. 2). Similarly, the Kornhall et al. study’s data point is located far to the left, demonstrating that intubation time was significantly shortened in the helicopter compared to the ground. The black dot on each line denotes the mean difference, and the black lines surrounding the dot represent the range of the 95% confidence interval, which is provided on the right. The RE model in the last line of the forest plot is one method used to unify all the mean difference values. The funnel plot shares the same RE model value as its black line in the middle, representing the standard error on the y-axis and the mean difference on the x-axis (Fig. 3). This plot also contains three data points with the same x-position as those in the forest plot. However, the y-position in the funnel plot includes the information provided in the confidence interval. In our analysis, the I^2^ statistic was found to be 99.65%, indicating a high level of heterogeneity among the included studies. This suggests that the true effect size may differ across studies, and caution is needed when interpreting the pooled results.

### Measured variables in the systematic review: results and interpretation

The systematic review included a total of three studies, of which two were comparative and one was retrospective. The primary outcomes assessed in these studies were the first pass success rate or total success rate, with the studies taking place in various settings including the BK-117, BO-105 helicopter, and EC135. The population in Harrison et al. [19] and Maeyama et al. [20] studies consisted of human participants, while Stone et al. [21] conducted their study on mannequins. The intubators involved in the studies included specialist physicians and flight nurses. The findings showed that the first pass success rate was notably lower in the helicopter group compared to the ground group (Table 4). However, the final success rate was relatively similar in both groups, indicating that additional attempts were successful in most cases. It is worth noting that variables like anthropometrics and daylight vs. night, reclination, noise and vibration, the expertise of the staff, and different scenarios of ETI may have influenced the results and should be taken into consideration when interpreting the findings of these studies.

**Table 4:** Description of the studies included in the systematic review: First pass success and total success rate on Helicopter vs. on ground

Author, year	Sample size (*n*=)	First pass success rate on Helicopter (%)	First pass success rate on Ground (%)	Total success rate Helicopter (%)	Total success rate Ground (%)
Harrison et al., 1997	303	75,0	79,7	94,2	98,3
Stone et al., 1994	30	N.A	N.A	93,0	100,0
Maeyama et al., 2020	376	88,5	93,5	98,4	97,3
Total	709	82,4	87,3	96,3	97,8

### Complications reported in the human studies

From a clinical perspective, the studies conducted by Harrison et al. and Maeyama et al. offer valuable insights into the complications observed during human trials. In the Harrison et al. study, a significant challenge arose in confirming tube placement, particularly in the context of helicopter noise, which hindered the exclusion of oesophageal intubation. To address this, the researchers employed various techniques, including monitoring chest movement, assessing end-tidal carbon dioxide flow, and observing tube fogging, as parameters for confirmation [22].

On the other hand, Maeyama et al. reported two primary complications: hypoxia and hypotension. Interestingly, they found no discernible difference in the incidence of these complications between ground-based and helicopter-based intubations [20]. These findings shed light on the complexities and challenges associated with intubation procedures in different settings, providing valuable insights for clinical practice and further research.

## Discussion

### Clinical implications

This study has a multifaceted clinical implication. First, the findings suggest that endotracheal intubation success rates are relatively comparable between helicopter and ground transport settings, despite higher rates of initial intubation failure in the helicopter settings. This implies that helicopter transport remains a viable option for critically ill patients requiring intubation during transport. However, it is important to note that the success rates of endotracheal intubation may be influenced by various variables such as anthropometrics, time of day, noise and vibration, and expertise of staff, among others. To the best of our knowledge, no previous studies identified in the literature or included in this systematic review have specifically investigated the potential influence of sound levels, vibration, confined space, anthropometric characteristics of the intubator, or other factors on endotracheal intubation success rates in the helicopter or ground transport settings. Combes et al. reported that the incidence of difficult ETI in a pre-hospital setting amounts to around 7.4%, independently of cardiorespiratory status. Later in 2015, Sunde et al. observed that cardiac arrest patients had a higher first-pass failure, in comparison to non-cardiac arrest patients [23]. Adverse events related to ETI in HEMS – i.e., hypotension, hypoxemia, or bradycardia- have been documented in several studies, but with highly trained personnel on board the incidence rate seems to be lower [7, 24, 25].

In addition to these factors, Knapp et al., also reported direct solar irradiation on the screen, fogging of the lens, and blood on the camera to significantly impair the first pass success [26]. Therefore, healthcare providers should be aware of these factors and take them into account when making decisions about transport options for critically ill patients.

The inconsistent results of the studies included in this meta-analysis regarding the time required for endotracheal intubation in helicopter and ground settings suggest that more research is needed to clarify the impact of various factors on intubation times. This could help intubators to better plan and allocate resources during transport, potentially leading to improved patient outcomes.

In addition to conducting statistical analysis, it is crucial to provide a practical context for our findings within the realm of prehospital care. The insights derived from the Maeyama study emphasize the importance of timing in the intubation process, particularly during patient transport, as a key factor influencing overall scene and prehospital durations [20]. The total pre-hospital time difference between the flight group (33,5 min) and the ground group (40 min) resulted to be statistically significant with *p* < 0,001, which also translates to a quicker patient treatment at the hospital.

Implementing intubation procedures during patient transport represents a promising strategy to expedite care delivery by reducing both scene and total prehospital times. The decision to perform intubation en route, as opposed to before departure, may play a critical role in optimizing time management within prehospital environments. Therefore, it is essential to consider not only the physical setting of intubation (inside or outside the vehicle) but also the temporal dynamics and decision-making processes involved in these procedures.

Consequently, while our analysis reveals variations in intubation times across different settings, it is crucial to recognize the pivotal role of intubation timing and its integration into broader prehospital care paradigms. Furthermore, this calls for a compelling need to investigate the nuanced factors that underpin successful endotracheal intubation within varying transport contexts. Healthcare providers should also be aware of the potential impact of various variables on intubation success rates and take them into account when deciding on transport options for critically ill patients. This holistic approach will contribute to improving patient outcomes and enhancing the efficiency of prehospital care systems.

### Contextualizing airway management in helicopters: the significance of simulated environments in research

We omitted the study conducted by McHenry and colleagues (2020) from our meta-analysis due to unavailability of data regarding the median duration of intubation, which serves as the primary parameter for our meta-analysis [22]. Instead, we were only able to obtain information on the Time to Secure Airway. While the duration of intubation is indeed a crucial factor, it is worth noting that Time to ETI Confirmation is equally significant. The Time to ETI confirmation encapsulates the duration spanning from the decision to intubate to the secure confirmation of endotracheal tube (ETT) placement, signifying a multifaceted journey. This metric traverses crucial stages, from patient positioning and equipment preparation to preoxygenation, drug administration, ETT insertion, confirmation of proper placement, and subsequent tube securing. However, a limitation in analyzed studies is the overemphasis on ETT insertion, neglecting the holistic intubation process complexity. Future research should adopt a comprehensive approach to scrutinize all intubation procedures, enhancing understanding and research validity. Given that the majority of other studies primarily utilized the duration of intubation, we selected it as the primary point of comparison for our meta-analysis.

Interestingly, Kornhall et al. also examined time to secure airway and reported a median time of 138 s for the standard group and 201 s for the Helicopter cabin. This indicates a substantial increase of nearly 50% in the required time compared to the McHenry study. In contrast, McHenry’s study found that the impact of being in the helicopter was relatively minimal. It resulted in a median increase from 231 s to 233 s for easy intubations, and a decrease of 7 s from 355 s to 348 s for hard intubations. This study provides insights into the physical barriers of rapid sequence intubation in-flight, as well as the factors affecting the cognitive capacity and situational awareness of crews. The results of this study can partially be overlapped with those of the two studies included in our meta-analysis, which showed minimal differences -or no difference-in intubation time between ground and helicopter transport. This study offering unique insights had a low Rob2 bias level, indicating a low risk of bias.

### Anthropometrics and reclination

The anthropometrics of both the intubator, including their height and weight and the patient’s head reclination are crucial factors to consider in assessing the success rates of endotracheal intubation (ETI) in prehospital settings. Several studies have demonstrated that the size and shape of a patient’s airway can impact the success of ETI, as well as the type of device used [27]. Moreover, the angle of the patient’s head and neck, in combination with the degree of stretcher reclination, can influence the positioning of the airway and the success rate. These factors are of relevance in helicopter transport, where space restrictions may require patients to be positioned differently. Therefore, it is essential to account for these variables when evaluating success rates and duration of ETI in various transport settings.

While the studies included in our analysis did not investigate the effect of these variables on intubation outcomes, it is plausible that they may influence the success rates of intubation in different settings. Given the potential significance of these variables, it would be beneficial for future research to examine their impact on intubation success rates in greater detail.

### Aircraft-helicopter type and location

Thomas et al. made a comparison between AS365N2 Dauphin and BK-117, which revealed that the type of aircraft or helicopter may have an impact on the decision-making of the crew and the effectiveness of endotracheal intubation [28]. Furthermore, Shekhar et al. reported that the location of the patient within the transport vehicle could also affect the first-pass success of endotracheal intubation, with fixed-wing air ambulances and rotor-wing vehicles performing the best [29]. These findings suggest that multiple factors, including the type of aircraft/helicopter and the location of the patient within the vehicle, should be considered when making decisions about endotracheal intubation during transport.

#### The expertise of the staff

The presence of expert staff in helicopter emergency medical services is crucial and has a significant impact on the success of endotracheal intubation and the duration of the procedure.

Peters et al. reported that HEMS staffed by physicians had a higher ETI success rate compared to those staffed by paramedics. They reported a significant different first-pass success rate of 46,4% in paramedics vs. 84,5% in HEMS physician group (*p* < 0.0001) [30]. . The Dutch researchers found that the average number of intubations per paramedic per year was less than three, which they believed accounted for the notable discrepancy between the groups. Gellerfors et al. conducted a study in 2018 and found that physicians had a significantly higher total success rate for tracheal intubation compared to nurses (99.0% vs. 97.6%; *p* = 0.03) [31].Overall, the success rate for endotracheal intubation appears to be higher when performed by experienced physician anaesthetists or nurse anaesthetists.

Furthermore, the introduction of physician-staffed HEMS appears to have expanded access to advanced prehospital care that was previously unavailable to patients. According to Sonne et al., the proportion of patients receiving interventions increased from 24.3 to 36.1% following the implementation of this service [32].

In this paper, most of the included studies had trained flight nurses or physicians as intubators but no subgroup analysis was made for lack of data.

### Environmental and other factors

None of the studies so far has analysed the impact of vibration, noise, confined space, or light on the quality of ETI.

Other factors might also affect this procedure, notably, Helm et al. and Naito et al. have reported that vomit, blood, and secretions in the upper airway can make laryngoscopy more difficult in HEMS settings [2, 33].

By analysing these questions, we hope to improve the safety, efficiency, and quality of care for patients who require pre-hospital endotracheal intubation, while promoting standardization of protocols and procedures in both HEMS and GEMS. Our findings may have important implications for clinical practice and could help improve patient outcomes in the critical pre-hospital care setting.

#### Limitations and strength

The study has some significant limitations that must be considered when interpreting the results. Firstly, the quality of the studies included was moderate due to limitations in study design and heterogeneity of the studies. Furthermore, the study had limited sample sizes and inconsistent methodologies across samples. Moreover, some important variables that may affect intubation success rates and duration were not consistently reported or controlled for in the studies included in the analysis. These include anthropometrics, reclination, noise and vibration, as well as the experience and expertise of the staff involved.

The majority of studies, including all the randomized controlled trials (RCTs), utilized mannequin models for simulating intubation scenarios. While these mannequin studies offer controlled environments for data collection, they inherently lack the complexity and variability of real-world intubation situations. Mannequin studies cannot fully replicate the dynamic challenges encountered during actual intubations, such as the presence of vomitus, deteriorating patient physiology, or unexpected airway obstructions.

Furthermore, the use of mannequin models may limit the generalizability of our findings to real clinical practice settings. Despite efforts to simulate realistic conditions, mannequin studies cannot fully capture the nuances and complexities of intubating patients in diverse clinical environments. As such, caution should be exercised when extrapolating the results of our meta-analysis to real-world patient care scenarios.

While our meta-analysis aimed to provide a comprehensive synthesis of available evidence, it is possible that relevant studies were inadvertently omitted or not included in our analysis. Variability in study methodologies, settings, and participant characteristics may also introduce heterogeneity and potential bias into our findings.

Despite these limitations, the study provides a valuable snapshot of the current literature and highlights the need for further high-quality studies to more fully explore the factors that impact intubation success rates and duration in different transport settings.

## Conclusions

Our systematic review and meta-analysis have provided important insights into the differences in endotracheal intubation success rates and mean duration of intubation in helicopter and ground transport settings. The findings suggest that while the first pass success rate of intubation is more likely to fail in the helicopter setting compared to the ground, the final success rate is relatively close. The studies analysed varied in their methodologies and population, highlighting the need for further research to fully explore the impact of variables such as anthropometrics, time of day, reclination, noise and vibration, and expertise of staff on intubation success rates in different settings.

The moderate quality of evidence underscores the limitations of the included studies, which could be influenced by potential biases and limitations in study design. It is imperative to conduct more high-quality studies to better understand the factors that contribute to successful endotracheal intubation in different transport settings.

This meta-analysis findings also suggest that endotracheal intubation times in helicopters and ground settings may vary significantly depending on the study design and population. The results of the three included studies were inconsistent, with Kornhall et al. reporting faster intubation times in helicopters, Gellerfors et al. finding no significant difference, and Lepa et al. reporting longer intubation times in helicopters. However, the larger sample size and higher quality score of the Lepa et al. study make it the most reliable and valid source of data. This inconsistency of results among the three included studies highlights the need for further research to identify factors that contribute to differences in intubation times.

In conclusion, this systematic review and meta-analysis highlight the need for continued research into the impact of various variables on endotracheal intubation success rates and time of intubation. This will help identify the factors that contribute to successful intubation in different transport settings and inform the development of best practices for clinicians performing endotracheal intubation.

## Data Availability

Data sharing is not applicable to this article as no datasets were generated or analysed during the current study. Any data or material can be given upon request.

## Abbreviations

DOI: Duration of Intubation
ETI: Endotracheal intubation
ER: Emergency Room
GEMS: ground emergency medical services
HEMS: Helicopter Emergency Medical Services
MD: Mean Differences
RCTs: Randomised Control Trials
